# The Waters of Contrexéville as a Cure for Certain Arthritic Conditions

**Published:** 1895-11

**Authors:** Debout D’Estrees

**Affiliations:** Paris


### THE WATERS OF CONTREXÉVILLE AS A CURE FOR CERTAIN ARTHRITIC CONDITIONS.

By DR. DEBOUT D'ESTREES, of Paris.

IN AMERICAN cities the tendency to arthritis is so frequent in the upper classes that, from a medical point of view, it may be regarded as one of their distinguishing characteristics.

Generally hereditary, this diathesis is kept up by the peculiar every-day life and hygienic conditions of those who live expensively. Let us consider the offspring of this class and follow the various phases of his life. As soon as the child can eat, he is overfed; at school he is overdriven intellectually whilst taking insufficient physical exercise. Later on, social obligations render it necessary for this same subject, now an adult, to take a daily share in dinners, which are so many infringements of the hygienic diet he ought to follow. Afterward, during the period of digestion, he goes to the theater, to evening parties, to his club, where he sits up late in an overheated atmosphere, where the air is vitiated, and at a time when his system is craving for oxygen to carry off, by the process of combustion, the effects of overfeeding.

The urine is overcharged, highly colored, and a deposit is oftentimes noticed in it; the patient is quite surprised by the unexpected advent of the first nephritic colic. The constant strain on the nervous system, the artificial diet peculiar to large towns, render oxalic gravel a frequent complaint. This oxalate of lime diathesis, so common in all parts of Europe and a special subject of study in the vast experimental field of Contrexéville, where persons so afflicted make up the great majority of patients, give rise to hematuria, to those intense dyspeptic troubles so frequently met with in America, to nervous phenomena which put an entirely new complexion on the character of the case.

Of the calculi, those composed of oxalate of lime are the hardest, the roughest and most pointed externally, and are the most likely to tear the mucous membrane during an attack of nephritic colic; they are always found to be stained with blood when definitely expelled *per vias naturales;* or if they form a stone in the bladder, lithotrity is powerless to break them up.

The urico-oxalic diathesis may likewise appear in the form of gout. Instead of symptoms of gravel, or even concomitant with them, an attack of gout occurs, first in the great toe, then in the hands; there is a certain amount of deformation and, possibly, tophi and nephritis, which is always ready to attack the gouty subject with unsparing severity.

Now, the Contrexéville waters are peculiarly adapted to the treatment of these cases; it is simply necessary that the patient should pay reasonably strict attention to diet and hygiene, to keep in a fair condition of health. This, unfortunately, by reason of the universal habit in America of subordinating everything to the demands of business and the rushing which prevails in social as well as commercial circles, is at the present epoch a hopeless prescription.

The physician who treats an arthritic subject is asked to keep his patient in a degree of health that will permit him to transgress many of the laws primarily essential to the maintenance of both nervous and physical systems in satisfactory working order. It is in this class of cases that the Contrexéville-Pavillon water is invaluable in guarding the kidneys from nephritis, the bladder from stone, and the joints from gouty deposits, by eliminating through the kidneys, day by day, the organic waste, the accumulation of which in the economy gives rise to the grave disturbances enumerated above.

Dr. Pâtissier, a member of the Paris Academy of Medicine, in a report read before that distinguished body, familiarly described this water as the "friend of the stomach." It certainly has a prompt and decided action in relieving acute attacks of hyperchlorhydric dyspepsia and periods of digestive inactivity.

My plan of treatment in a condition of arthritic diathesis has been to order the patient to drink a bottle of Pavillon water before breakfast, a glass at intervals of twenty minutes, with an interval of from three-quarters to one hour between the last glass and the first meal, this treatment to be continued until the morbid condition is rectified. The water is easily and rapidly absorbed, and, when taken in the morning, prepares the stomach for the rapid digestion of the daily meals and keeps up the freedom of the intestinal functions. It can also, and most advantageously, be mixed with wine, which it does not render turbid, like alkaline waters, and to which it does not give any special taste. Thus the diuretic action will be continued and completed, while the peristale of the digestive tract is sufficiently strong to ensure facility of digestion and the daily regularity of the alvine evacuations.

It must be borne in mind, however, that this practice does not do away with the necessity of the true method of treatment with this water, when prescribed in nephritic colic or acute albuminuria, resulting from continued uric acid diathesis, which consists in the absorption, during twenty consecutive days, every morning, on an empty stomach, of a bottle of Contrexéville-Pavillon water, one glassful to be drunk every twenty minutes, one half hour to elapse between the last glassful and the first meal.

